# Fly Ash-Based Geopolymer Building Materials for Green and Sustainable Development

**DOI:** 10.3390/ma13245699

**Published:** 2020-12-14

**Authors:** Rosicky Methode Kalombe, Victor Tunde Ojumu, Chuks Paul Eze, Sammy Mwasaha Nyale, John Kevern, Leslie Felicia Petrik

**Affiliations:** 1 Department of Chemical Engineering, Cape Peninsula University of Technology, P.O. Box 1906, Bellville, Cape Town, Western Cape 7535, South Africa; OjumuT@cput.ac.za; 2 Department of Civil and Mechanical Engineering, University of Missouri-Kansas City, 5110 Rockhill, Kansas City, MO 64110, USA; kevernj@umkc.edu; 3 Department of Chemistry, University of the Western Cape, Private Bag X17, Bellville 7535, South Africa; ceze@uwc.ac.za (C.P.E.); lpetrik@uwc.ac.za (L.F.P.); 4 Water and Environment Unit, Council for Geoscience, 280 Pretoria Street, Silverton, Pretoria 0184, South Africa; smwasaha@gmail.com

**Keywords:** fly ash, geopolymer, compressive strength, carbon dioxide, construction

## Abstract

This study reports on formulations and conditions for producing fly ash-based geopolymers with a view to showing that the compressive strength required for construction applications can be obtained without the addition of aggregates, sand, and/or cement. It was shown in a series of experiments constituting at least 73% fly ash that a compressive strength of up to 90 MPa can be obtained depending on the curing conditions. While high alkalinity resulted in stronger materials, the results showed about 40% savings in CO_2_ emissions without using sand and cement. Such materials are suited for construction applications with minimal environmental impact.

## 1. Introduction

Concrete is the second most used material after water in the world [1]. The main component of concrete is ordinary Portland cement (OPC). It is known that the production of cement is highly energy-intensive, next to steel and aluminum, and that it consumes an important amount of natural resources such as limestone. As the limestone is heated, it changes into lime and CO_2_. These CO_2_ emissions represent 60 to 65% of total emissions linked to cement production [2,3]. Thus, for every ton of OPC produced, approximately one ton of CO_2_ is emitted, depending upon the method used [4]. In 2016, cement plants were described as emitting around 2.2 billion tons of CO_2_ into the atmosphere [5]. For these reasons, it is necessary to develop new kinds of concretes, called green concretes. 

As the global population increases, so does the need for power. Although many carbon-neutral energy sources exist, coal is widely available and cheap. Coal fly ash is generated as a by-product of coal combustion, and globally, the volume of waste fly ash produced is increasing. Millions of tons of coal ash are produced worldwide. From 2016 to 2017, South African coal-fired power plants produced about 32.6 billion tons of coal ash [6]. Dry fly ash from improperly maintained ash piles poses a health risk if inhaled because of the very small particles (less than 10 µm diameter). Beneficiation of coal fly ash could save vast amounts of land otherwise lost to ash ponds and landfills while minimizing potential pollution and liability from these sources. 

Fly ash has found applications in the treatment of acid mine drainage [7,8] and the synthesis of zeolites [9,10,11]. Its abundant availability and pozzolanic properties create the opportunity to utilize it as a substitute for cement in the production of concrete. Yao et al. [12] reported that coal fly ash can be successfully used in construction. The binder could be produced by a polymeric reaction of alkali liquids with silicon and aluminum available in coal fly ash; these binders together are termed “geopolymer” [13].

In 1979, Davidovits used the term geopolymer for the first time to describe a class of three-dimensional aluminosilicate materials produced from sources like clay, red mud, and fly ash [14]. The use of fly ash in the synthesis of geopolymer takes advantage of the naturally high concentration of SiO_2_ and Al_2_O_3_ [15]; low SiO_2_ and Al_2_O_3_ content is not adequate for alkali-activation [16]. Typically, ASTM C618 Class F fly ash with lower calcium is preferable than the higher calcium Class C fly ash [17]. The difference between the two classes of fly ash is based on the sum of total aluminum, silicon, and iron oxides in the ash. When the sum is greater than 70%, the fly ash is termed Class F, whereas when the sum is between 50% and 70%, the ash belongs to Class C [18]. Class C fly ashes show poor reactivity with alkaline activators due to their low glass content and high calcium content [19].

Fly ash-based geopolymers are formed through alkali activation of fly ash, which is the acceleration of the dissolution reactions, utilizing activators. Various activators such as NaOH, KOH, Na_2_SiO_3_, and K_2_SiO_3_ have been used in the synthesis of geopolymer, either singly, or in combination, as the alkaline liquids to be added to fly ash [20,21]. NaOH solution has been observed to provide a higher level of dissolution of minerals compared to KOH, and thus, it yields better geopolymer performance [22,23]. Kaur et al. [21] indicated that approximately 1.2 times more compressive strength could be obtained when a combination of NaOH and Na_2_SiO_3_ was added to fly ash, compared to the use of NaOH only. The compressive strength increased as NaOH concentration increased from 8 to 16 M. Thus, the increase of NaOH concentration caused a significant acceleration of dissolution reactions, promoting the early stages of the geopolymerization process [24,25]. A maximum compressive strength of 40.42 MPa was obtained when fly ash was activated with 16 M NaOH and mixed with a sand to fly ash ratio of 3:1 [21]. However, Singh et al. [26] and Wattimena et al. [17] reported the highest compressive strength when 14 M NaOH was used during geopolymer synthesis. Slag, coarse sand, and aggregates were used as fillers in the geopolymer to increase the strength. Rattanasak and Chindaprasirt [27] investigated the effect of the concentrations of 5, 10, and 15 M NaOH. The highest compressive strength, i.e., up to 70 MPa, was attained when the mixture was formulated with 10 M NaOH, a ratio of Na_2_SiO_3_/NaOH of 0.1:1, and a sand/fly ash ratio of 2.75:1. Furthermore, Sathonsaowaphak et al. [28] and Kaur et al. [21] reported that high compressive strength was developed in a range of 0.45–0.75:1 ratio of the alkaline activator to bottom ash or fly ash.

The other factor that impacts the geopolymer synthesis outcome is the water content. Patankar et al. [29] indicated that minimizing the water content will increase the NaOH concentration in the aqueous phase. However, the geopolymer reaction requires the presence of enough moisture to develop good strength [30]. Davidovits [31] stated that the curing time required for developing geopolymer products varied from 6 to 100 h (h), and that the optimal temperature was below 100 °C. Two curing temperatures of 60 and 80 °C have mostly been used in the literature [20,21,30,32,33,34,35]. The curing temperature and aging period not only play a significant role as accelerators of the chemical reactions, but also determine the extent of those reactions [30,36]. The aging periods mostly used to test the compressive strengths of geopolymer materials are 7 and 28 days [21,25,37,38,39,40,41,42].

Several authors have investigated the synthesis of geopolymers with fly ash for different purposes and applications. For instance, Hamid et al. [43] reported that a geopolymer could potentially enhance some of the properties of the asphalt binder. Wang and Zhao [32] demonstrated that a dosage of 25 wt% of fly ash or slag was crucial to obtain excellent flame retarding efficiency. Nath [25] developed a fly ash and zinc slag (40−80 wt %) blended geopolymer for the immobilization of hazardous materials and paving blocks. Furthermore, fly ash-based geopolymer spheres were evaluated as a methylene blue adsorbent material by Novais et al. [44]. It should be highlighted that the dominant reaction product of the geopolymer process consisted of sodium-containing aluminosilicate hydrogel (N-A-S-H gel) [45].

The properties of geopolymers are very similar to OPC when formed under appropriate conditions [46]. However, there is a strong difference between Portland cement hydration and alkali activation of fly ash [24,25]. Alkali activation is similar to the chemistry involved in the synthesis of large groups of zeolites [11]. Geopolymers can be used in the building and construction industries to make many products, including bricks and roof tiles. However, fly ash-based bricks and roof tiles must satisfy the acceptable standard for durability, which should be taken into account as the major test of the quality in cases where roof tiles are used for building roofs, or bricks are used for water reservoirs, basements, and tunnels [23,25,47].

As mentioned above, geopolymer products made with mixtures of coal fly ash, in combination with alkaline activators, aggregates, or other aluminosilicate materials have been reported so that good mechanical properties could be developed. The development of new formulations for preparing geopolymer materials with good mechanical properties that do not involve the addition of aggregates or other aluminosilicate materials to coal fly ash is thus an area of research that necessitates further attention. Therefore, this research aims to develop new formulations for the synthesis of geopolymer materials with good mechanical properties that do not involve the addition of fillers or other aluminosilicate materials to coal fly ash. In addition, this research will explore the effect of additional water, in conjunction with various concentrations of NaOH, as well as physical parameters such as the curing temperature and aging period during the synthesis of fly ash-based geopolymer. Then, the amount of CO_2_ released in the atmosphere by cement or fly ash materials will be compared in order to propose the most environmentally friendly building material.

## 2. Materials and Methods

### 2.1. Raw Materials

In this study, fly ash from a coal-fired power station in Mpumalanga (Republic of South Africa) was utilized. The alkaline activator used was a combination of NaOH and Na_2_SiO_3_. The NaOH was in pellet form with 98% purity, and the Na_2_SiO_3_ in liquid phase consisted of SiO_2_ = 30%, Na_2_O = 9.2%, H_2_O = 60.8%. Additional deionized water was also used.

### 2.2. Characterization of the Raw Material

Coal fly ash was analyzed for use in the synthesis of geopolymer materials. Coal fly ash was collected downstream of the electrostatic precipitators or bag filters. The fly ash samples were kept in sealed plastic containers away from sources of moisture, and these containers were stored in a dark, cool cupboard to protect them from any temperature variations. The mineral phase, compositions of the major elements, and morphology of coal fly ash were identified using X-ray diffraction (Philips PANalytical instrument with a pw3830 X-ray generator, Bruker, Coventry, UK), X-ray fluorescence (Philips 1404 Wavelength Dispersive spectrometer, Bruker, Coventry, UK), and scanning electron microscope (LEO SEM 1450, ZEISS, Germany), respectively.

### 2.3. Preparation of Materials and Experimental Procedure

NaOH solutions of 10, 12, 14, and 16 M were utilized. An alkaline activator comprising a combination of Na_2_SiO_3_ and NaOH solution (for each molar concentration of NaOH) was prepared just before mixing with fly ash and water to ensure the reactivity of the solution.

The workability was enhanced with the addition of either an extra amount of water to the fly ash or a more concentrated alkaline activator solution, which resulted in a homogeneous paste. The mass ratios of an extra amount of water to fly ash of 0.1, 0.075, 0.05, and 0.025 were varied, and all other parameters were kept constant. The extra amount of water was first mixed with the alkaline activator (Na_2_SiO_3_ solution and specified concentration of NaOH solution mixed for 30 min); then, this solution was mixed with fly ash [27] in the mixing unit made of a plastic tank and a mechanical stirrer rotating at a speed of 55 rpm in order to form a homogeneous paste. The pastes were then poured into molds (size: 100 mm^3^) painted with a high viscosity oil as the releasing agent.

The molds were wrapped with plastic sheeting in order to keep the moisture in the samples, and then cured in an oven at 60 or 80 °C for 24 h. Then, all the geopolymers were taken out of the molds, covered with plastic sheeting, and kept at room temperature for the rest of the required curing time, i.e., 7 and 28 days. 

### 2.4. Test Variables

Table 1 shows the four conditions investigated during the synthesis of fly ash-based geopolymer. Each condition consisted of variable and constant parameters. The variable parameters were water/fly ash ratios, molar concentrations of NaOH, curing temperatures, and the aging periods, as shown in Table 1.

Approximately 64 experimental formulations were developed for geopolymer synthesis using the mix proportions depicted in Table 1. Therefore, for each ratio of water to fly ash (0.1, 0.075, 0.05, or 0.025), every concentration of NaOH (10, 12, 14 or 16 M), curing temperature (60, or 80 °C), and aging period (7, or 28 days) was used to synthesize geopolymer materials in this study.

All the conditions (see Table 1) were simultaneously investigated. For instance, for a water to fly ash ratio of 0.1:1, the experiments were investigated by varying different parameters in the following order: (i) a water to fly ash ratio of 0.1:1 was mixed with the alkali liquid, 10 M of NaOH was used, and the mixture was then cast in the molds. The geopolymer paste was cured in an oven at 60 °C for 24 h and aged for 7 days. (ii) The geopolymer paste was prepared in the same way as in (i) but aged for 28 days. (iii) The geopolymer paste was prepared in the same condition as in (i) but cured in an oven at 80 °C for 24 h and aged for 7 days. (iv) The geopolymer paste was prepared in the same condition as (iii) but aged for 28 days. Other experiments were performed as described from (i) to (iv), but the fly ash was activated with 12, 14, or 16 M of NaOH during the synthesis of geopolymer materials.

In this section, an explanation was given for the experiments carried out using a water to fly ash ratio of 0.1:1 and the same procedure was followed using water to fly ash ratios of 0.075:1, 0.05:1, and 0.025:1. Also, all the conditions or formulations used for the synthesis of geopolymer materials were carried out in triplicate in the present study. 

### 2.5. Compressive Strength

The compressive strength tests were performed on the geopolymer samples aged 7 and 28 days in accordance with “Compressive Strength on concrete cubes” SANS method 5863:2006. An Automatic Max testing machine [King test auto 2000 model Pat 2001 with load capacity and load rate of 2000 kN and 40–1000 kN/min, respectively, Cape Laboratory equipment (Pty), Republic of South Africa] was used to determine the ultimate strength of the geopolymer. The samples were subjected to a load of 180 kN, and the loading rate was 180 kN/min for a sample of a dimension of 100 mm^3^. The reported compression strength values were an average of the results obtained for the three samples produced for each condition and formulation.

### 2.6. Water Absorption

A water absorption test was performed on the geopolymer product samples obtained after 28 days of aging, according to the ASTM D570-98 standard [48]. The test was performed to determine the amount of water absorbed by a geopolymer under the stated conditions. The water absorption test was performed using geopolymer samples made of ratios of water/fly ash of 0.025:1, Na2SiO3/NaOH of 2.5:1, NaOH/fly ash of 0.1:1, NaOH solutions of 10, 12, 14, and 16 M, cured in an oven at 60 °C for 24 h and aged for 28 days. The samples were weighed and then put in a bucket full of water (at approximately 23 °C) for 24 h. Thereafter, the samples were removed, patted dry with a lint-free cloth, and weighed. Thus, the unit of water absorption is in weight percent. 

### 2.7. Production of Fly Ash-Based Roof Tiles and Paving Bricks

The roof tiles and paving bricks were produced at a lab-scale using the condition described in Table 1, with a ratio of water/fly ash of 0.025:1, NaOH/fly ash of 0.1:1, and Na_2_SiO_3_/NaOH of 2.5:1. The geopolymer paste was cast in the molds and cured at 60 °C for 24 h.

The fly ash-based roof tiles and paving bricks were manufactured by activating coal fly ash with 10 M of NaOH. The mechanical properties and durability developed at this concentration during the optimization studies met the standards for building materials. These standards are SANS 542-2012 for concrete roof tiles and SANS 1058-2012 for concrete paving blocks [49,50].

### 2.8. Estimation of the Quantity of CO_2_ Emitted by Cement and Fly Ash Products

A comparison was made between construction bricks made of fly ash and cement in order to quantify the CO_2_ embodied by these bricks in the environment when used in the construction of houses. The mix formulation used is as shown in Table 2.

It can be shown that the total number of bricks required for the construction of a two-room structure with dimensions 18.7 m length and height of 2.25 m [49] is 7086 bricks [53]. Therefore, the amount of CO_2_ emitted due to the use of these materials for brick making can be calculated using the emission estimated of CO_2_ per kilogram of materials, as shown in Table 3. 

## 3. Results and Discussion

### 3.1. Characterization of Coal Fly Ash

Table 4 shows the distribution of the major elements present in three samples of coal fly ash, as determined by XRF.

Table 4 indicates that the fly ash characterized using XRF belongs to Class F [18], as the total amount of silicon, aluminum, and iron oxide was 91.16 wt %, while the composition of calcium oxide was 4.49 ± 0.07 wt %. The XRF analysis also showed that the mass ratio of SiO_2_ to Al_2_O_3_ was 1.85, with silica being the most abundant compound. The ratio of Si to Al was 1.57:1; according to Panias et al. [60], this ratio determines the formation and application of geopolymer materials.

**Figure 1:**
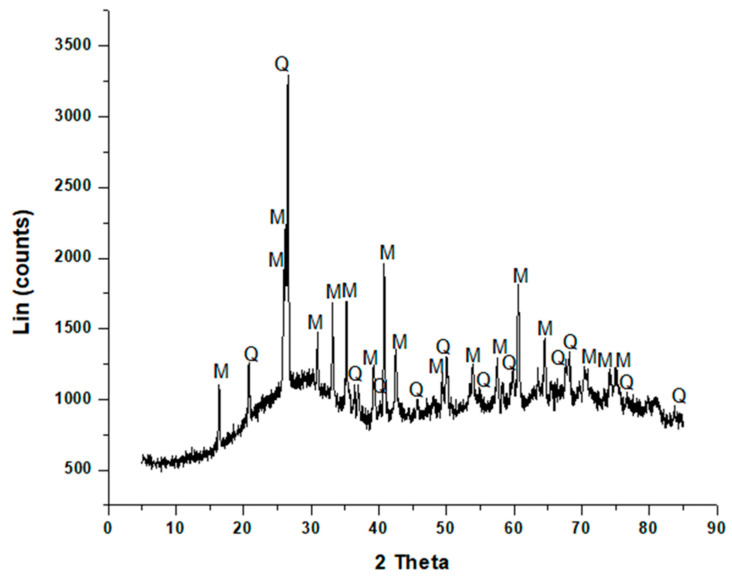
Qualitative XRD of coal fly ash. Q = quartz, M = mullite.

Therefore, this type of fly ash can be used in the synthesis of geopolymer, because it is preferable to prepare geopolymers using ASTM Class F fly ash with low calcium rather than Class C with high calcium content, the reason being that the Class C fly ash setting time during the geopolymer synthesis is very short, and the material tends to flash set in some cases [17]. The mineral phases identified in the sample of fly ash by XRD are shown in Figure 1.

Two major phases were identified in fly ash, namely, quartz (SiO_2_) and mullite (3Al_2_O_3_2SiO_2_), as shown in Figure 1. The XRD spectrum showed a broad hump occurring between 17° 2θ and 38° 2θ, which may be attributed to the amorphous glassy phase contained in fly ash [61]. 

The mineral composition was that of a low calcium coal fly ash which was also highly amorphous, with the presence of a small amount of quartz and mullite, demonstrated by low-intensity diffraction peaks. The major mineralogy of fly ash was amorphous glass, which was important, because it bestowed pozzolanic properties upon the fly ash [62].

Therefore, there was consistency between the elemental analysis obtained from XRF and the different phases obtained from XRD data, which revealed that the glass phase, as well as quartz and mullite, contained a high percentage of silica and alumina. The observations made about coal fly ash using XRD were consistent with those of other authors [40,63,64].

**Figure 2:**
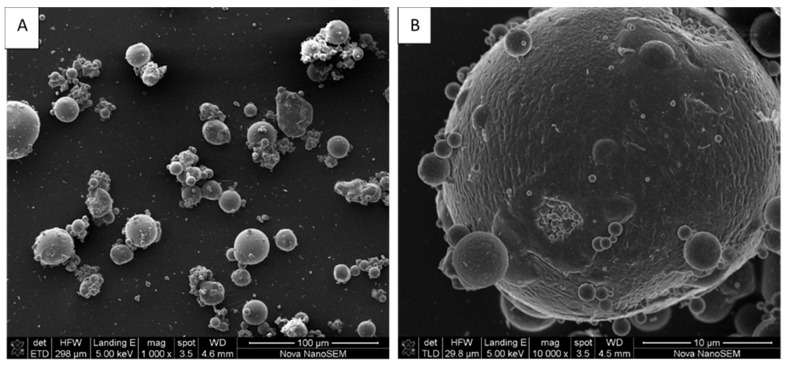
SEM micrographs of coal fly ash particle sizes were taken at the magnification of (**A**) 1000×, and (**B**) 10,000×.

It is always important to know the phase composition in fly ash, because the amorphous glassy phase content is one of the reactive components that can be efficiently used to form a geopolymer product at mild temperatures, whereas quartz and mullite require fusion at high temperatures for the release of Si and Al content [65]. The morphology of coal fly ash, as observed from scanning electron microscopy measurements, is shown in Figure 2. Using the “ImageJ software”, the coal fly ash was shown to have a mean particle size of 6.10 µm with a minimum of 1.34 µm and a maximum of 14.31 µm. It has been reported in the literature that fly ash particle size ranges between 0.1 µm and 200 µm [66,67].

As depicted in Figure 2A,B, the SEM images were obtained using the same fly ash sample but at different magnifications. The particles of fly ash were spherical with some being closely attached, forming agglomerates. This could be a consequence of thermochemical transformations of mineral particles in the high–temperature coal combustion process, through which the minerals melt to produce small droplets, which, upon rapid cooling and by the action of surface tension forces, adopt a spherical shape, occurring as small grains. In addition, the smooth appearance of the outer surface of the coal fly ash particles can be attributed to the presence of aluminosilicate glassy phases [68].

### 3.2. Compressive Strength

**Figure 3:**
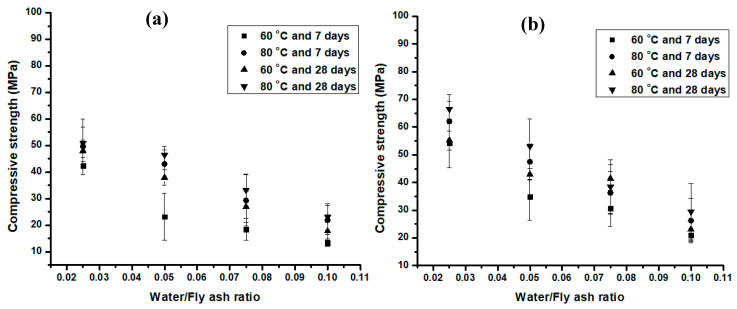

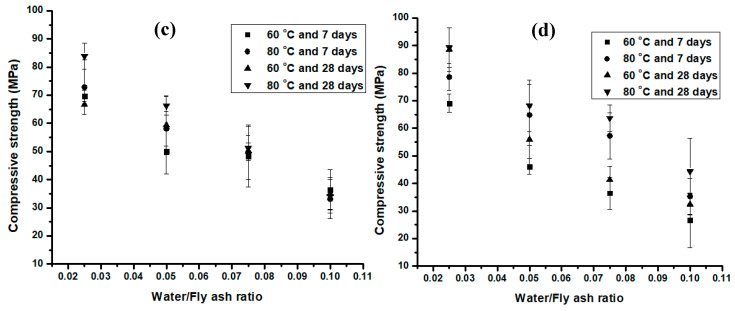
Compressive strength resulting when adjusting different parameters involved in the synthesis of geopolymer, (**a**) 10, (**b**) 12, (**c**) 14 and (**d**) 16 M of NaOH (Fixed parameters were ratio of Na_2_SiO_3_/NaOH = 2.5 and NaOH/fly ash = 0.1 as described in Section 2).

Figure 3 shows compressive strengths developed by the fly ash-based geopolymer with aging times of 7 and 28 days.

Figure 3 demonstrates that the compressive strengths of a series of geopolymers varied from 13.39 ± 1.42 MPa to 89.32 ± 7.1 MPa. It was observed that the strengths increased with an increase in NaOH concentration from 10 M [Figure 3a] to 14 M [Figure 3c]; but statistically, there was not much difference in strength between the samples activated with 14 and 16 M [Figure 3d] of NaOH. Statistically, there was not much difference in strength between geopolymers cured at 60 and 80 °C for 24 h. Similar observations were made for samples aged 7 and 28 days. Hence, shorter times and lower temperatures were sufficient for adequate strength development, which required less energy, making them more feasible.

As shown in Figure 3, the water content had a significant effect on the hardening process of geopolymer. The compressive strength decreased with an increase in the amount of water. This could be attributed to many factors including; (i) in the geopolymer, water is not bound in the reaction products, but acts as a transport mechanism for the dissolution of silicate and alumina to occur. (ii) The increase in water content results in the reduction of NaOH molar concentration which, in turn, affects the high dissolution of silicon and aluminum ions that takes place in the high molarity of the NaOH solution. Na and OH are important during the geopolymerization process because Na ions can balance the charges of the aluminosilicate networks that act as the binder in the mixture [20]. Moreover OH^−^ improves the rate of dissolution of the glassy phase. The level of dissolution is dependent on the composition as well as the alkali concentration of the mixture [69]. Only a small amount of water is required for the workability of the alkali-activated coal fly ash [29,42]. The flow of the geopolymer paste increased with an increase in the water to geopolymer ratio, but an excess of water had an impact on its strength development. The geopolymer reaction requires the presence of moisture to develop good strength [20,70]. However, the compressive strength of the geopolymer product decreased with an increase in water content, as shown in Figure 3. 

**Figure 4:**
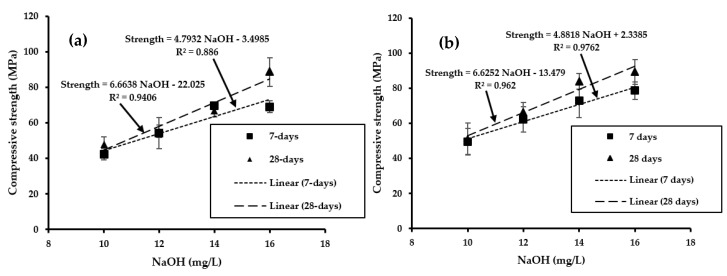
Regression model obtained using the compressive strength versus NaOH concentration of geopolymer cured at (**a**) 60 °C, and (**b**) 80 °C (fixed parameters of the ratio of water/fly ash of 0.025:1, Na_2_SiO_3_/NaOH of 2.5:1, NaOH/fly ash of 0.1:1, and aged of 7 days and 28 days).

Based on the results in Figure 3, it was observed that a water/fly ash ratio of 0.025:1 produced the strongest geopolymer. Thus, Figure 4 shows only the strengths developed by geopolymer samples made of a ratio of water/fly ash of 0.025:1, NaOH/fly ash of 0.1:1, and Na_2_SiO_3_/NaOH of 2.5:1, at different molar concentrations of NaOH, cured at 60 and 80 °C, and aged for 7 and 28 days.

Figure 4a,b shows the linear regression obtained from the formulation and conditions developed in this study. R-squared values measured how much of the variation in strength was explained by NaOH molarities in the regression model. Thus, the higher the R-squared value, the better the regression model fitted the data. The compressive strengths and NaOH molarities investigated in this study correlated and could be used to predict the compressive strength of the fly ash-based geopolymer at different curing temperatures or ages. It could be said that the compressive strength of geopolymer was a function of the NaOH concentration, which, in turn, depended upon the water content.

Figure 4a,b demonstrates how the increase in NaOH concentration produced high compressive strength. This was attributed to a significant acceleration of the dissolution reactions of alumina and silica in fly ash caused by the increase in NaOH concentration, which promoted the early stages of the geopolymerization process [15]. These parameters enhanced the dissolution of fly ash, resulting in the increase of Al and Si concentrations in the aqueous phase, which improved the condensation process of Si and Si-Al oligomer formation. Thus, the process of oligomer polycondensation enhanced the hardening of the geopolymeric structure [17,26,27,60].

The geopolymer produced through the reaction of fly ash only with an alkaline activator could be considered as an alternative to the utilization of ordinary Portland cement in the construction industry [12,20]. These results implied that in the manufacturing of products using the conditions and formulations developed in this study, the products could be made and used within 7 or 28 days, because they would have a compressive strength of up to 90 MPa.

Based on the above results, the novelty of this study consisted of: (1) the developed formulations, and (2) the final product quality and characteristics, and its new application in the construction industry. These formulations could be a feasible remediation for several problems, including:

- The utilization of coal fly ash as the main source of silica, alumina, and lime, thus reusing this waste and resolving the disposal problem of coal-fired power station plant waste.
- No need for the addition of cement or aggregates to the geopolymer, thus reducing the mass and the carbon footprint of cement manufacturing
- No need for high temperature kilning
- Replacement of cement with coal fly ash in the construction industry
- Minimum addition of alkaline activators
- Reduced cost of geopolymerization process

The compressive strengths reported in Figure 3 and Figure 4 were higher than those reported in the literature by many authors [17,21,26,27]. This could be attributed to the difference in experimental procedures, mix proportions, or the properties of the raw materials (including alkali liquid) utilized. In this study, the coal fly ash had very fine spherical particle sizes which constituted the major factor affecting the reactivity of the fly ash and the amount and compositions of the amorphous or glassy phases. This had an impact on the condensation of sodium-containing aluminosilicate hydrogel, which is important for the formation of the three-dimensional aluminosilicate network [45].

However, many fly ash-based geopolymers previously reported in the literature for different purposes and applications could be improved by applying the formulations developed in this study. These formulations could be used to improve the fly ash-based geopolymer coating for fire resistance materials [32], the immobilization of hazardous substances [25] and backfilling materials [71], low micro-filling effect (low porosity) materials [24], absorbent materials [44], or asphalt binders [43].

### 3.3. Water Absorption of Geopolymer Products

Table 5 shows the water absorption and compressive strength of the resultant geopolymer products after the paste had been activated with different NaOH concentrations. These samples were made using a ratio of water/fly ash of 0.025:1, Na2SiO3/NaOH of 2.5:1, and NaOH/fly ash of 0.1:1, cured in an oven at 60 °C for 24 h, and aged for 28 days, after which their compressive strengths and water absorption (as described in Section 2.6) tests were evaluated. 

Table 5 shows that both water absorption and compressive strength data were related to NaOH concentration. These results indicated that the increase in alkalinity during geopolymerization tended to decrease the water absorption of the resultant product. High alkaline formulations with low water content produced a less porous and stronger structure of the final geopolymer product, with low water penetration. Geopolymer activated with high NaOH concentrations resulted in lower water absorption. Low water absorption was ascribed to low porosity, which increased the strength of the fly ash-based geopolymer and affected the pore size of the geopolymer material. Higher porosity occurred when a high amount of water was used in the mixture because the inorganic polymer produced large gel crystals with trapped water inside. As soon as the water evaporated from the pores during curing at 60 °C for 24 h, the product became more porous, resulting in a higher percentage of water absorption and low compressive strength, because strengths deteriorated when the evaporation of moisture occurred [72].

### 3.4. Production of Fly Ash-Based Roof Tiles and Paving Bricks

**Figure 5:**
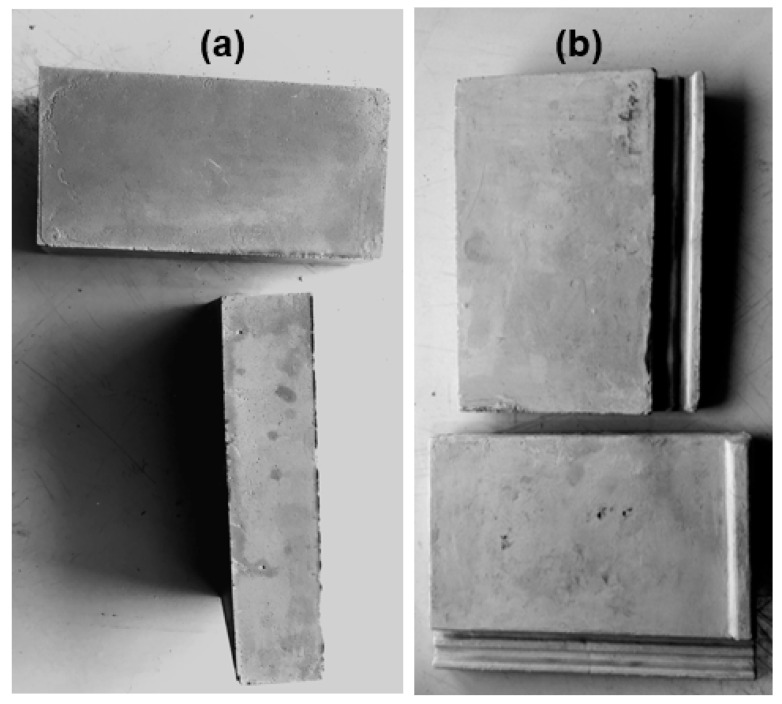
Fly ash-based products, (**a**) paving bricks, and (**b**) roof tiles.

Figure 5 shows the paving bricks and roof tiles made in this study. These products were made as described in Section 2.7.

Figure 5a,b shows the paving bricks and roof tiles produced in this study. The average masses of three samples of paving bricks and roof tiles were 2.13 ± 0.10 and 3.33 ± 0.15 kg, respectively. The dimensions of the paving bricks were 220 mm in length, 110 mm in width, and 49 mm in depth, while those of the roof tiles were 293 mm in length, 214 mm in width, and 32 mm in depth.

The water absorption for the formulation used to make the paving bricks and roof tiles was 7.55 ± 0.17%. It could be said that the paving bricks made in this study met the compressive strength and water absorption requirements for commercial paving brick because, according to SANS 1058:2012 for concrete paving blocks, there are two classes, namely class 30, which indicates a compressive strength of 30 MPa, and class 40, with a compressive strength of 40 MPa. The water absorption should fall within the range of 6.5% and 8% [49]. Therefore, the standards were met for paving bricks made using the geopolymer formulations and conditions developed in this study. The durability and mechanical properties of fly ash-based paving bricks can compete with concrete and clay paving bricks available in the market. Also, fly ash-based roof tiles met the SANS 542-2012 standard for concrete roofing.

The paving bricks and roof tiles were made at a laboratory scale and no fillers, pigments, fibers, or other compounds were added; however, such compounds may be added in future to make the bricks and tiles suitable for use as commercial roof tiles or paving bricks. Thus, the aim of making the roof tiles and paving bricks from fly ash was to demonstrate that with the appropriate equipment (i.e., molds), many building materials, molded art objects, and ceramics [31] can be manufactured using the formulations and conditions developed in this study, without the need for additional cement, sand, aggregate, or other fillers.

### 3.5. Quantification of CO_2_ Emission from Cement and Fly Ash Products 

It has been previously shown that 7086 bricks are required for the construction of a two-room structure (see Section 2.8). For the following calculations, only CO_2_ corresponding to the material composition was included; it was assumed that the amount of CO_2_ emitted during the manufacturing and transportation of either brick type would be the same; therefore, manufacturing and transportation have been excluded from the calculations for this example. Using the information provided in Table 3, it follows that over 21,258 kg of fly ash could be diverted from landfill, compared to about 5397 kg of cement for the construction of a two-room structure, with a consequent saving of 1391 kg of carbon dioxide (Table 6).

According to Totaro [73], the number of houses in the informal settlement in the Endlovini Township part of Khayelitsha, Cape Town (Republic of South Africa) is about 6600. For this number of houses, it can be inferred that about 46.7 million bricks would be required to construct two-room structures in the modernization of the township, with a potential saving of 9179 tons of CO_2_ if fly ash (24,058 tons of CO_2_ embodied) were used in place of cement (33,236 tons of CO_2_ embodied) for this purpose. This would utilize about 140,303 tons of fly ash or 35,623 tons of cement to make the fly ash or cement bricks, respectively.

Therefore, it can be said that utilization of fly ash instead of cement to make building materials would reduce the amount of CO_2_ and other emissions into the atmosphere from cement production by decreasing the need for limestone calcination, thus minimizing the consumption of fossil fuels, as well as reducing sand and aggregate mining, which is also environmentally damaging.

## 4. Conclusions 

The present study resolved many problems. Utilizing coal fly ash as the main source of silica, alumina and lime makes it possible to reuse this waste and resolves the disposal problem of coal-fired power station plant waste. As there was no need for the addition of cement or aggregates to the geopolymer, the formulations reduced the carbon footprint caused by cement manufacturing. Mild curing conditions eliminated high-temperature brick kilning. Geopolymerization offers a route for the replacement of cement with coal fly ash in the construction industry. The formulations developed herein reduced the addition of alkaline activators. 

The significance of this study may be summarized as follows:

1. No coarse aggregate, sand, or cement was used in any of the formulations developed in this study. The compressive strengths of a series of experiments varied from 13.39 ± 1.42 to 89.32 ± 7.1 MPa. The increase in alkalinity during geopolymerization tended to decrease the product’s water absorption, which varied from 3.74 ± 0.78 to 7.55 ± 0.17%, indicating low water penetration.
2. The best formulation and condition applied for the synthesis of a geopolymer with high strength of 89.32 ± 7.1 MPa consisted of a ratio of water/fly ash of 0.025:1, 16 M of NaOH, 80 °C curing temperature, and an aging time of 28 days.
3. The fly ash-based roof tiles and paving bricks produced in this study had a compressive strength and water absorption of 47.98 ± 4.15 MPa and 7.55 ± 0.17%, respectively, meeting SANS 1058:2012 and SANS 542-2012 specifications.
4. In order to produce bricks to build 6600 houses, cement materials produced a higher amount of CO_2_ compared to fly ash materials. A huge amount of fly ash was required compared to cement in order to produce the same amount of bricks, but a huge saving of sand was achieved. Approximately 140,303 tons of fly ash could be diverted from landfill and a reduction of 9179 tons of CO_2_ achieved when substituting fly ash bricks for conventional cement-based blocks in construction. Thus, these benefits help make fly ash an environmentally friendly choice for construction, with one of the lowest carbon footprints of any building material.

The findings of this study may change the classification of coal fly ash from waste to a resource that could be used in the manufacturing of various materials. So far, roof tiles and paving bricks are the only products to have been manufactured at the lab-scale. However, other building materials, ceramics, or art objects could be produced using the formulations and conditions developed in this research.

Future studies could focus on the investigation of other mechanical and durability properties of the optimum formulations, in order to find applications in the manufacturing of a variety of building materials. Also, identifying or developing fly ash-based geopolymers tailored to local specifics for the modernization of informal settlements worldwide should be explored in the future. 

## Figures and Tables

**Table 1:** Mix proportions per condition.

Condition	Variable	Constant
1	Water/fly ash (wt)	Constant
	0.1:1	NaOH/fly ash = 0.1:1, Na_2_SiO_3_/NaOH = 2.5:1, 60 °C, 7 days
	0.075:1	
	0.05:1	
	0.025:1	
2	NaOH (mg/L)	Constant
	10	Water/fly ash = 0.025:1, NaOH/fly ash = 0.1:1, Na_2_SiO_3_/NaOH = 2.5:1, 60 °C, 7 days
	12	
	14	
	16	
3	Temperature (°C)	Constant
	60	Water/fly ash = 0.025:1, NaOH/fly ash = 0.1:1, Na_2_SiO_3_/NaOH = 2.5, 7 days
	80	
4	Age (day)	Constant
	7	Water/fly ash = 0.025:1, NaOH/fly ash = 0.1:1, Na_2_SiO_3_/NaOH = 2.5:1
	28	

Note: all ratios are by weight (wt).

**Table 2:** Mix formulation one brick.

Cement Brick *		Fly Ash Brick	
Water/Cement	0.6:1	Water/Fly ash	0.1:1
Sand/Cement	6:1	NaOH/Fly ash	0.1:1
		Na_2_SiO_3_/NaOH	2.5:1

Note: NaOH concentration is 10 M, Na_2_SiO_3_ is in the liquid phase. * Source: [51,52].

**Table 3:** One kg of material per CO_2_ emission.

Material	CO_2_ Emission (kg)	Reference
Cement	0.87	[54]
Sand	0.01	[55]
Fly ash	0.012	[56]
Water	0.005	[57]
NaOH	0.5297	[58]
Na_2_SiO_3_ (37% liquid)	0.424	[4,59]

Note: 0.012 kg of CO_2_ consisted of the sum of emissions during the collection and transportation of fly ash.

**Table 4:** Distribution of fresh coal fly ash.

Major Oxides	A	B	C	Average
	wt %			
SiO_2_	57.17	56.68	56.61	56.82 ± 0.31
Al_2_O_3_	30.24	30.85	31.03	30.71 ± 0.41
Fe_2_O_3_	3.64	3.65	3.62	3.63 ± 0.01
CaO	4.50	4.55	4.42	4.49 ± 0.07
TiO_2_	1.61	1.61	1.59	1.60 ± 0.01
MgO	1.11	1.10	1.12	1.11 ± 0.01
K_2_O	0.71	0.72	0.67	0.70 ± 0.02
P_2_O_5_	0.51	0.50	0.50	0.50 ± 0.01
MnO	0.03	0.02	0.03	0.03 ± 0.01
Cr_2_O_3_	0.04	0.04	0.03	0.04 ± 0.01
Na_2_O	0.42	0.27	0.35	0.35 ± 0.07
V_2_O_5_	0.03	0.02	0.03	0.03 ± 01
Total	100.00	100.00	100.00	100.00 ± 0.00
SiO_2_/Al_2_O_3_	1.89	1.84	1.82	1.85 ± 0.04
Si/Al	1.61	1.56	1.55	1.57 ± 0.03

Note: wt % stands for weight percent, A B C stands for a different batch of coal fly ash.

**Table 5:** Water absorption of fly ash-based geopolymer products.

NaOH (M)	Compressive Strength (MPa)	Water Absorption (%)
10	47.98 ± 4.15	7.55 ± 0.17
12	55.21 ± 3.41	4.79 ± 0.20
14	66.64 ± 3.43	4.39 ± 0.83
16	88.59 ± 7.97	3.74 ± 0.78

**Table 6:** Cement or fly ash materials required for the construction of a two-room structure per quantity of CO_2_ emission.

Cement Brick			Fly Ash Brick		
Material		CO_2_ Emission	Material		CO_2_ Emission
(kg)			(kg)		
Cement	5397	4696	Fly ash	21258	255
Sand	32387	324	NaOH	2126	1126
Water	3238	16	Na_2_SiO_3_	5315	2253
			Water	2126	11
Total	41022	5036		30824	3645
